# Daylight saving time does not seem to be associated with number of percutaneous coronary interventions for acute myocardial infarction in the Netherlands

**DOI:** 10.1007/s12471-021-01566-7

**Published:** 2021-03-25

**Authors:** L. Derks, S. Houterman, G. S. C. Geuzebroek, P. van der Harst, P. C. Smits, the PCI Registration Committee of the Netherlands Heart Registration, G. Amoroso, E. K. Arkenbout, S. Aydin, J. Brouwer, C. Camaro, J. Daemen, P. W. Danse, M. van der Ent, R. Erdem, J. P. Henriques, A. W. J. van ’t Hof, I. Karalis, A. Kraaijeveld, J. P. van Kuijk, E. Lipsic, M. Margo, K. M. J. Marques, A. J. M. Oude Ophuis, J. van Ramshorst, V. Roolvink, W. T. Ruifrok, M. Scholte, C. E. Schotborgh, B. J. Sorgdrager, F. Spano, M. G. Stoel, T. Teeuwen

**Affiliations:** 1 Netherlands Heart Registration, Utrecht, The Netherlands; 2 Department of Cardiothoracic Surgery, Radboudumc, Nijmegen, The Netherlands; 3 Department of Cardiology, Division of Heart & Lungs, University Medical Center Utrecht, University of Utrecht, Utrecht, The Netherlands; 4 Department of Cardiology, Maasstad Hospital, Rotterdam, The Netherlands

**Keywords:** Circadian rhythm, Daylight saving time, Myocardial infarction, Netherlands, Percutaneous coronary intervention

## Abstract

**Background:**

In multiple studies, the potential relationship between daylight saving time (DST) and the occurrence of acute myocardial infarction (MI) has been investigated, with mixed results. Using the Dutch Percutaneous Coronary Intervention (PCI) registry facilitated by the Netherlands Heart Registration, we investigated whether the transitions to and from DST interact with the incidence rate of PCI for acute MI.

**Methods:**

We assessed changes in hospital admissions for patients with ST-elevation myocardial infarction (STEMI) or non-STEMI (NSTEMI) undergoing PCI between 1 January 2015 and 31 December 2018. We compared the incidence rate of PCI procedures during the first 3 or 7 days after the transition with that during a control period (2 weeks before transition plus second week after transition). Incidence rate ratio (IRR) was calculated using Poisson regression. Potential gender differences were also investigated.

**Results:**

A total of 80,970 PCI procedures for STEMI or NSTEMI were performed. No difference in incidence rate a week after the transition to DST in spring was observed for STEMI (IRR 0.95, 95% confidence interval (CI) 0.87–1.03) or NSTEMI (IRR 1.04, 95% CI 0.96–1.12). After the transition from DST in autumn, the IRR was also comparable with the control period (STEMI: 1.03, 95% CI 0.95–1.12, and NSTEMI: 0.98, 95% CI 0.91–1.06). Observing the first 3 days after each transition yielded similar results. Gender-specific results were comparable.

**Conclusion:**

Based on data from a large, nationwide registry, there was no correlation between the transition to or from DST and a change in the incidence rate of PCI for acute MI.

## What’s new?

- Previous studies have shown conflicting results for the relation between daylight saving time (DST) and the incidence of myocardial infarction (MI) or cardiac death.
- This is the first study investigating the impact of DST using a large, nationwide database containing of all percutaneous coronary intervention (PCI) procedures in the Netherlands.
- We found no relation between the transition to or from DST and the number of PCI procedures for acute MI.

## Introduction

In order to save energy, daylight saving time (DST) has been used continuously in the Netherlands since 1977 [1]. Moving the clock one hour forward in spring results in a delayed sunset and more daylight in the afternoon, until the clock is moved back in autumn. The practice of DST has been harmonised and is obligatory for all European member states since 2001 [2]. However, on 26 March 2019, the European Parliament approved a legislative resolution to discontinue the pan-European implementation of DST after May 2021 and leave implementation of DST up to the individual states [3, 4].

One of the arguments to end the biannual seasonal change of time is the potential health effects. In multiple studies, the influence of seasonal time changes and the effect of circadian rhythm disruption on health outcomes have been investigated. In particular the adaptation of the circadian rhythm after the clock change in spring appears to be problematic for some people and could result in adverse health outcomes [5, 6]. A meta-analysis by Manfredini et al. from 2019, which included 7 studies performed between 2012 and 2018, suggested there is an increase in the incidence of myocardial infarction (MI) after the transition in spring, but not in autumn [7]. Not all published study results have been univocal and outcomes may differ between countries based on geographical position. As such, the influence of DST is probably larger in Nordic countries than in Mediterranean countries [8].

Therefore, we investigated the potential relation of the transition to or from DST with the incidence of acute MI for which patients underwent percutaneous coronary intervention (PCI) in a large Dutch nationwide registration.

## Methods

### Study population

Since 2015, data on all performed PCI procedures by the 30 Dutch PCI centres are collected in the Netherlands Heart Registration (NHR). The NHR is a nationwide, physician-driven and patient-focused quality registry that contains procedural and outcome data of all cardiovascular interventions and surgery in the Netherlands. The purpose of the NHR is to be conducive and contribute to quality improvement of cardiovascular care in the Netherlands. For each intervention or operation, patient characteristics, indication for the intervention, procedural data and outcome data are collected and submitted to the NHR. For PCI procedures, this includes information on medical history regarding renal failure, diabetes mellitus, multivessel disease, chronic total occlusion, history of MI, cardiogenic shock, out of hospital cardiac arrest and previous coronary artery bypass grafting (CABG), among others. Definitions have been standardised and are available at the NHR website (www.nederlandsehartregistratie.nl/handboeken). Site-specific data are reviewed by each site, whereas the NHR performs independent and random data monitoring.

For this study, we selected all hospital admissions for patients (≥ 18 years) with ST-elevation myocardial infarction (STEMI) or non-STEMI (NSTEMI) undergoing PCI between 1 January 2015 and 31 December 2018. The week after the transition to DST in spring and the week after the transition from DST in autumn were marked as the period of interest. The control period consisted of the 2 weeks before each transition plus the second week after the transition. Furthermore, we compared the first 3 days after each transition with the control period and reviewed potential differences in incidence between men and women. Gender-specific analyses were performed to compare results with the international literature. In the Netherlands, DST starts on the last Sunday of March and ends on the last Sunday of October.

### Statistical analysis

Categorical variables are presented as number and percentage. Statistical significance was tested using the chi-squared test for categorical variables. Incidence rate ratio (IRR) with 95% confidence interval (CI) was calculated for the period of interest and the control period using Poisson regression. Observed incidence was the mean number of PCI procedures performed each day during the period of interest and the control period. *P*-values were corrected for multiple testing (false discovery rate) using a Benjamini-Hochberg correction [9, 10]. *P*-values < 0.05 were considered statistically significant. All statistical analyses were performed using IBM SPSS Statistics for Windows, version 26.0.

## Results

In the period 2015–2018, 161,727 PCI procedures were performed in the Netherlands. There were 80,970 PCI procedures with an indication of NSTEMI (42,873) or STEMI (38,097). A total of 12,751 PCI procedures (NSTEMI: 6748, STEMI: 6003) were performed during the period of interest or control period and were subsequently included for analysis. Demographics of the population are presented in Tab. 1. All patient characteristics were over 95% complete, except for renal failure (11% unknown).

**Table 1:** Demographic characteristics of patients with non-ST-elevation myocardial infarction (*NSTEMI*) or ST-elevation myocardial infarction (*STEMI*) treated with percutaneous coronary intervention in the Netherlands (2015–2018)

Variable	Study group (*n* = 12,751)	Intervention performed outside of study period (*n* = 68,219)	*P*-value
*Indication*			
NSTEMI	6748 (52.9)	36,125 (53.0)	0.945
STEMI	6003 (47.1)	32,094 (47.0)	
*Age, years*			
≥ 80	1723 (13.5)	9247 (13.6)	0.774
70–79	3284 (25.8)	17,549 (25.7)	
60–69	3593 (28.2)	19,350 (28.4)	
50–59	2773 (21.7)	14,960 (21.9)	
< 50	1378 (10.8)	7113 (10.4)	
*Gender*			
Male	9143 (71.7)	48,892 (71.7)	0.936
Female	3608 (28.3)	19,327 (28.3)	
*Renal function*			
eGFR ≥ 60	8821 (77.6)	47,092 (77.4)	0.748
eGFR 30–59	2238 (19.7)	12,075 (19.9)	
eGFR 15–29	227 (2.0)	1130 (1.9)	
eGFR < 15	58 (0.5)	322 (0.5)	
On dialysis	28 (0.2)	191 (0.3)	
Diabetes mellitus	2415 (19.5)	12,922 (19.5)	0.871
Multivessel disease	5891 (46.6)	31,511 (46.5)	0.669
Single vessel disease	6754 (53.4)	36,219 (53.1)	0.378
Chronic total occlusion	314 (2.5)	1557 (2.3)	0.255
History of myocardial infarction	2604 (21.0)	14,592 (22.0)	0.043
Cardiogenic shock	483 (3.8)	2580 (3.8)	0.471
Out of hospital cardiac arrest	674 (5.3)	3586 (5.3)	0.555
Previous CABG	942 (7.5)	4994 (7.4)	0.597

Data are *n* (%)

*eGFR* estimated glomerular filtration rate,* CABG* coronary artery bypass grafting

Regarding baseline characteristics, a history of MI was slightly less frequently seen in the study population (21%) than in patients who underwent an intervention outside the selected study period (22.0%, *p*-value 0.043). No other statistically significant differences between groups were observed. Similarly, the patient group undergoing PCI during the period of interest (DST group) was compared with the control group on baseline characteristics. No significant differences were observed (results not shown).

**Fig. 1:**
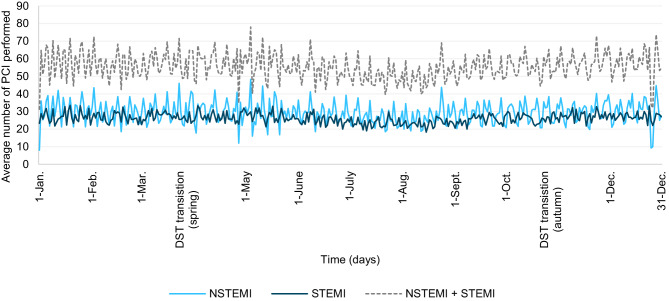
Mean number of percutaneous coronary interventions (*PCIs*) performed per day by indication over 4 years (2015–2018). *DST* daylight saving time, *NSTEMI* non-ST-elevation myocardial infarction, *STEMI* ST-elevation myocardial infarction

Fig. 1 shows the mean number of PCI procedures for STEMI and NSTEMI per day throughout the year, with a lower number of PCI procedures performed in the summer. The day-to-day fluctuation was less for PCI procedures for STEMI than for NSTEMI.

### One week after transition

Mean incidence rate and IRR of PCI procedures for STEMI or NSTEMI per day for the week following each transition are shown in Tab. 2, for the total population and separately by gender. Overall, no significant differences in incidence rate were observed in the week after the transition in both spring and autumn (see ‘Total’ in Tab. 2).

**Table 2:** Mean incidence rate and incidence rate ratio (*IRR*) of percutaneous coronary intervention procedure for ST-elevation myocardial infarction (*STEMI*) or non-STEMI (*NSTEMI*) 1 week after transition to daylight saving time (spring) and from daylight saving time (autumn) versus control period

	STEMI					NSTEMI				
Season	Period of interest (1 week)	Control period	IRR (95% CI)	*P*-value	Adjusted *p*-value	Period of interest (1 week)	Control period	IRR (95% CI)	*P*-value	Adjusted *p*-value
*Spring*	26.2	27.7	0.95 (0.87–1.03)	0.188	0.526	31.5	30.4	1.04 (0.96–1.12)	0.365	0.786
Men	18.6	20.2	0.92 (0.84–1.02)	0.111	0.444	22.5	22.2	1.01 (0.92–1.11)	0.817	1.000
Women	7.5	7.5	1.01 (0.86–1.18)	0.936	1.000	9.7	8.2	1.18 (1.02–1.36)	0.028	0.196
*Autumn*	26.9	26.1	1.03 (0.95–1.12)	0.503	0.880	29.2	29.8	0.98 (0.91–1.06)	0.630	1.000
Men	18.9	19.1	0.99 (0.90–1.10)	0.891	1.000	20.4	20.6	0.99 (0.90–1.09)	0.828	1.000
Women	7.9	7.0	1.13 (0.96–1.31)	0.134	0.469	8.8	9.3	0.95 (0.82–1.10)	0.480	0.816
**Total**										
Spring	57.6	58.0	0.99 (0.94–1.05)	0.807	1.000					
Autumn	56.0	55.9	1.00 (0.95–1.06)	0.913	1.000					

Data are incidence rate per day

*CI* confidence interval

For PCI procedures for STEMI patients, the incidence rate per day in the week after the transition in spring or autumn was comparable to that in the control period (spring: 26.2 vs 27.7, autumn: 26.9 vs 26.1) and the IRR was not significant for spring or autumn. Observed mean incidence rate per day was lower for women than for men in both spring and autumn. None of the observed IRRs was significant after correction for multiple testing (Tab. 2).

For PCI procedures for NSTEMI patients, the IRR a week after the transition in spring or autumn was not significant. Stratified analysis in men and women revealed a higher IRR for women in spring (1.18, 95% CI 1.02–1.36), but the ratio was not significant after correction for multiple testing. Overall incidence rate per day was lower for women than in men (Tab. 2).

### Three days after transition

Tab. 3 shows mean incidence rate and IRR of PCI procedures for STEMI or NSTEMI per day for the 3 days following each transition, also for men and women separately. Overall mean incidence rate per day was lower in the first 3 days after the start of DST in spring (52.6) than during the control period (58.0), resulting in an IRR of 0.91 (95% CI 0.83–0.98). After correction for multiple testing, the incidence rate in the 3 days after the transition did not differ significantly from the control period, neither in spring nor in autumn (see ‘Total’ in Tab. 3).

**Table 3:** Mean incidence rate and incidence rate ratio (*IRR*) of percutaneous coronary intervention procedure for ST-elevation myocardial infarction (*STEMI*) or non-STEMI (*NSTEMI*) 3 days after transition to daylight saving time (spring) and from daylight saving time (autumn) versus control period

	STEMI					NSTEMI				
Season	Period of interest (3 days)	Control period	IRR (95% CI)	*P*-value	Adjusted *p*-value	Period of interest (3 days)	Control period	IRR (95% CI)	*P*-value	Adjusted *p*-value
*Spring*	27.0	27.7	0.98 (0.87–1.10)	0.676	1.000	25.6	30.4	0.84 (0.75–0.95)	0.005	0.140
Men	19.8	20.2	0.98 (0.85–1.12)	0.750	1.000	18.9	22.2	0.85 (0.74–0.98)	0.022	0.205
Women	7.3	7.5	0.97 (0.77–1.21)	0.778	1.000	8.0	8.2	0.97 (0.77–1.22)	0.971	0.971
*Autumn*	27.4	26.1	1.05 (0.94–1.18)	0.408	0.816	26.2	29.8	0.88 (0.78–0.99)	0.032	0.179
Men	20.4	19.1	1.07 (0.94–1.23)	0.316	0.804	18.5	20.6	0.90 (0.78–1.03)	0.137	0.426
Women	7.0	7.0	0.99 (0.79–1.25)	0.954	0.989	7.7	9.3	0.83 (0.66–1.02)	0.082	0.383
**Total**										
Spring	52.6	58.0	0.91 (0.83–0.98)	0.019	0.266					
Autumn	53.6	55.9	0.96 (0.88–1.04)	0.323	0.754					

Data are incidence rate per day

*CI* confidence interval

Stratified analysis of the 3 days after the transition to DST in spring by indication for PCI yielded a lower IRR for NSTEMI, but not for STEMI. Further stratification of the results showed a lower IRR for NSTEMI in men (0.85, 95% CI 0.74–0.98), but not in women (0.97, 95% CI 0.77–1.22). These results indicate a lower number of PCI procedures in the 3 days after the transition to DST (spring) than in the control period. However, after correction for multiple testing, none of the observed IRRs was significant (Tab. 3).

The IRR in the following 3 days after transition from DST in autumn for PCI procedures for STEMI patients was similar to that during the control period (Tab. 3). However, a lower IRR was observed for PCI procedures for NSTEMI patients (0.88, 95% CI 0.78–0.99). Further stratification of the results for NSTEMI yielded similar results for men and women. None of the observed IRRs was significant after correction for multiple testing.

## Discussion

Even though we observed fluctuations in the mean number of PCI procedures performed per day around the transition to or from DST, none of the IRRs was significant after correction for multiple testing. Therefore, this study based on Dutch data did not show an increased number of PCIs for acute MI in the week or first 3 days after the transition. There were also no differences between men and women.

Shortening or lengthening the ‘day’, which is the result of implementing DST, impacts the circadian rhythm of the human body. Severe disruption of this circadian rhythm is associated with multiple negative health outcomes and potentially increases the risk of adverse cardiovascular events. Several research groups have provided explanations for the adverse effects due to DST, which have been summarised in an ex-post evaluation of DST by the European Parliamentary Research Service [1] and a review by Meira et al. [6].

The adverse effect of DST on health may be influenced by the body’s ability to cope with circadian disruption [6]. This ability is related to an individual circadian preference (chronotype) [11]. People with an extreme chronotype experience more profound effects of DST on sleep disruption and a potential adverse effect of this disruption on their health [12]. Studies on chronotype distribution around the globe report differences between countries, gender and age categories [13, 14]. Since chronotype is a likely confounder for the relation between the potential effect of DST on human health, country-specific analyses and stratified analyses by gender or age should be made to effectively study this potential effect.

Based on their meta-analysis, Manfredini et al. concluded a relatively modest significant increase in MI risk in the week after the spring transition to DST, with no substantial differences between genders [7]. Five of the included studies were carried out in a European country [8, 15–18]. We did not find any new studies focusing on DST and MI in Europe. Culic [17] and Janszky et al. [16, 18] reported a significantly higher incidence of acute MI in the first 3 days or first week after the transition in spring. In contrast, our uncorrected results yielded a lower mean incidence rate of PCI for NSTEMI in the first 3 days after the transition in spring. The previous studies had included all (hospitalised) patients with acute MI, whereas only acute MI patients in need of PCI were included in the current study. Our results provide no indication for an increase in incidence of severe and acute MIs.

After correction for multiple testing, our findings were not statistically significant and were in line with what has been reported in more recent studies performed by Kirchberger et al. [8] and Sipila et al. [15]. Since it has been indicated that the distribution of chronotypes in a population varies according to geographical location, we only compared our results with studies performed in Europe. Nevertheless, none of these studies, including our own, was able to investigate chronotype distribution, which potentially confounds the relation between DST and acute MI and therefore could explain the difference in results.

Lindenberger et al. investigated the influence of DST on cause of death in more detail and showed a higher number of autopsy cases in the first week after the transition to DST [19]. The higher mortality rate was caused by a higher number of suicides, not cardiovascular deaths. In the current study, the number of PCI procedures for STEMI or NSTEMI in the period of interest and the control period was comparable. A rise in cardiovascular deaths due to coronary artery disease is therefore not to be expected.

As far as we know, this is the first study assessing the potential effect of DST on the incidence rate of PCI for acute MI in the Netherlands. For this study, data from the nationwide database of the NHR were used. This database contains detailed and complete information of all performed PCI procedures in the Netherlands. It was therefore possible to identify a large cohort of patients with clinically relevant MI. Furthermore, our statistical analyses are comparable with previous studies [15–18, 20, 21], meaning we identified a similar reference period and calculated the IRR with Poisson regression. Additionally, we corrected for multiple testing. When performing multiple tests, the likelihood of falsely detecting an effect (type I error) increases. The Benjamini-Hochberg correction lowers this risk but is still effective in identifying a true effect [9, 10].

### Limitations

Except for patients with STEMI or NSTEMI in need of PCI, patients treated with CABG, medically treated patients with acute MI and prehospital coronary deaths were not included in the study. Furthermore, due to the retrospective design of our study, no data on sleep duration, sleep quality or chronotype were available. Due to the lack of statistical power, stratified analyses were only performed for men and women. Further stratification of patients by medication use or medical history, as proposed by others [8, 16], could not be performed. Finally, it was not possible to derive causality between the observed and described associations.

## Conclusion

Our study did not show an increase in hospital admissions for patients with an acute MI (STEMI/NSTEMI) in need of PCI around DST in the Netherlands. In light of the European Parliament’s resolution to abolish pan-European DST and leave the decision to individual states, country- and region-specific studies are essential to decide which DST strategy is best for each member state. In these studies, the economic impact should be weighed against the potential negative health effect of DST, knowing that no effect on the number of PCIs for an acute MI was observed in the Netherlands.
